# Influence of Different Evolutive Forces on *GDF5* Gene Variability

**DOI:** 10.3390/genes14101895

**Published:** 2023-09-30

**Authors:** Laura Flore, Paolo Francalacci, Myosotis Massidda, Renato Robledo, Carla Maria Calò

**Affiliations:** 1 Department of Life and Environment Sciences, University of Cagliari, 09042 Cagliari, Italy; laura.flore@unica.it (L.F.); paolo.francalacci@unica.it (P.F.); cmcalo@unica.it (C.M.C.); 2 Department of Medical Sciences and Public Health, University of Cagliari, 09042 Cagliari, Italy; myosotis.massidda@unica.it; 3 Department of Biomedical Sciences, University of Cagliari, 09042 Cagliari, Italy

**Keywords:** single nucleotide polymorphism, Population Branch Statistic, selective pressure

## Abstract

The *GDF5* gene is involved in the development of skeletal elements, synovial joint formation, tendons, ligaments, and cartilage. Several polymorphisms are present within the gene, and two of them, rs143384 and 143383, were reported to be correlated with osteoarticular disease or muscle flexibility. The aim of this research is to verify if the worldwide distribution of the rs143384 polymorphism among human populations was shaped by selective pressure, or if it was the result of random genetic drift events. Ninety-four individuals of both the male and female sexes, 18–28 years old, from Sardinia were analyzed. We observed the following genotype frequencies: 28.72% of AA homozygotes, 13.83% of GG homozygotes, and 57.45% of AG heterozygotes. The allele frequencies were 0.574 for allele A and 0.426 for allele G. The relationships between the populations were verified via Multidimensional Scaling (MDS). Our data show (i) a clear heterogeneity within the African populations; (ii) a strong differentiation between the African populations and the other populations; and that (iii) the Sardinian population is placed within the European cluster. To reveal possible traces of selective pressure, the Population Branch Statistic (PBS) was calculated; both the rs143384 and 143383 SNPs have low PBS values, suggesting that there are no signals of selective pressure in those areas of the gene.

## 1. Introduction

Growth factor differentiation 5 (GDF5) is a protein that has received particular interest, since its mutations, in studies carried out both in mice and in humans, are related to defects in the appendicular skeleton or to abnormal joint development [1]. The interest in this protein is corroborated by a large number of studies. In particular, during the past year, 36 papers were published on this topic (PubMed, accessed on 23 July 2023), with 14 of them dealing with humans.

This protein, which belongs to the transforming growth factor β (TGF-β) superfamily, is encoded by the *GDF5* gene. Members of this family regulate the cell growth and differentiation of several tissues at both the embryonic and adult stages. 

*GDF5* is one of the first genes that is expressed in the embryonic joint interzone, where it is involved in the development of skeletal elements [2]. It contributes to accelerating the initial stages of chondrogenicity and thus controls the proliferation and differentiation of chondrocytes [3,4,5]. It also participates in the formation of the synovial articulation, tendons, ligaments, and cartilage, their maintenance, as well as in bone formation [6]. 

Furthermore, GDF5 is a neurotrophic protein that has effects on dopaminergic neurons [1] and regulates the growth of neuronal axons and dendrites, carrying out a role in inflammatory response and tissue damage [7]. 

Accordingly, mutations of the *GDF5* gene have been associated with several pathologies caused by skeletal defects or abnormal joint development observed in congenital diseases, including Hunter-Thompson-type and Grebe type chondrodysplasia [8]. 

*GDF5* gene mutations are also responsible for type C brachydactyly, which is characterized by the underdevelopment or absence of phalanges and metacarpals [9]. In addition, genome-wide association studies (GWASs) have suggested that *GDF5* gene polymorphisms are associated with knee and hip arthrosis [10,11]. 

Special attention was given to the association between *GDF5* and osteoarthritis (OA) since it represents the most common form of arthritis, which is associated with aging, and affects more than 50% of people over 65 years old, regardless of sex and ethnicity [12]. OA is a multifactorial disease characterized by the progressive destruction of articular cartilage, synovial inflammation, and bone remodeling that leads to pain and the loss of joint function, especially in the knees, hips, and hands [13]. Clinical studies demonstrated that, in OA, joint integrity is impaired by an imbalance between extracellular cartilage matrix (ECM) degradation and synthesis controlled by chondrocytes [14].

The gene, which includes four exons, has been mapped on chromosome 20 at position q11.22. Among the numerous polymorphisms present in the *GDF5* gene, eight Single Nucleotide Polymorphisms (SNPs) have been identified through GWAS analysis (www.gwascentral.org). The most studied SNPs are rs143383 and rs143384, both located in the 5′ UTR region at a distance of 227 bp (see Table 1), which turned out to be risk factors for osteoarthritis in adults [15]. In particular, an analysis of the rs143383 SNP indicated that the T allele (corresponding to the A allele in NCBI and the 1000 Genomes dataset) showed reduced transcriptional activity and is overrepresented in patients with osteoarthritis [16]. Likewise, the C allele of rs143383 (corresponding to the G allele in NCBI and the 1000 Genomes dataset) is a protective factor against the susceptibility to knee osteoarthritis [17] and stress fractures [18]. 

It was hypothesized that epigenetics has a role in *GDF5* expression. Precisely, it was observed that the CpG sites created by the C alleles at rs143383 and rs143384 were variably methylated, and the demethylation caused an increase in *GDF5* expression. On the contrary, the counterpart alleles caused a reduction in the *GDF5* promoter sequence activity, and consequently, a reduction in the *GDF5* in human cartilage [13].

In the same way, it was hypothesized that the G allele of rs143383 could protect against a potential injury of the lower limbs [19]. A recent study by Meng et al. [20] on the whole genome suggested that rs143384 is associated with knee pain and the A allele represents the predisposing factor. More recently, rs143384 was identified as being significantly associated with an increased susceptibility to chronic postsurgical pain, again, with the A allele carriers showing an increased risk [7]. However, the contribution of the *GDF5* gene has not been directly elucidated based on biological evidence. 

In a different study [21], the allelic variants of the *GDF5* gene, which included rs143383 and rs143384, were shown to be associated with congenital dislocation of the hip (CDH) in Caucasians. The most significant association was observed with the rs143384 SNP. Individuals who are homozygous for the T allele (corresponding to the A allele in NCBI and the 1000 Genomes dataset) have a higher risk of developing CDH compared to carriers of the other two genotypes. 

In terms of the other six SNPs, evidenced by the GWAS analysis (reported in dbSNP as rs224330, rs224331, rs224332, rs224333, rs224334, and rs7267783), no strong evidence of an association between specific alleles and clinical conditions has been reported in the literature, and therefore, they were not further investigated in this report. 

There is an increasingly frequent use of the regulatory region of the *GDF5* gene in association studies to check its putative association with osteoarticular diseases. Therefore, the present research aims to verify if the worldwide distribution of gene polymorphisms among human populations, particularly for the most investigated SNP, rs143384 (G-A), was shaped by selective pressure or if it was the result of random genetic drift events. 

## 2. Materials and Methods

For the present research, we analyzed the genetic variability of SNP rs143384 in 2598 individuals. Most of the individuals (2504) belong to 26 populations from all continents, and the sample was obtained from the 1000 Genomes public database. Furthermore, we increased the sample size by adding new data from Sardinia (Italy) by sampling 94 individuals of both sexes (53 females and 41 males) who were apparently healthy, unrelated, and born in Sardinia or have been residents of Sardinia for at least three generations.

The research was performed in agreement with the Declaration of Helsinki for Human Research of 1974 (last modified in 2000), and each participant signed a written informed consent form. The study was approved by the Ethics Committee of the Azienda Ospedaliera Universitaria (AOU) of Cagliari University (Italy). 

A buccal swab was obtained from each participant and preserved in absolute ethanol. DNA was then extracted using the salting-out method. DNA concentration and purity were determined using a NanoDrop spectrophotometer (Thermo Scientific, Waltham, MA, USA). The following primers were designed with PRIMERS BLAST (accessed on 4 June 2022): forward: 5′-AGGCAGCATTACGCCATTC-3′; reverse: 5′-TGAATTCCAGGTCCAGCCAA-3′. DNA was amplified in a 25 μL mixture containing 200 ng of genomic DNA, 20 pmol of each primer, and 0.2 U/μL of NZYTaq II (NZytech, Lisbon, Portugal). PCR protocol consisted of an initial denaturing step at 94 °C for 5 min, followed by 30 cycles at 94 °C for 30 s, 53 °C for 45 s, and 72 °C for 1 min, with a final extension step at 72 °C for 5′. Amplicons were sequenced with Sanger method by Macrogen—Italy Service (Milan, Italy). Allele and genotype frequencies, as well as Hardy–Weinberg equilibrium, were calculated with Genepop (ver. 4.4.3). Population variability was analyzed using the 1000 Genome dataset (https://www.internationalgenome.org/ (accessed on 20 June 2023)); matrix genetic distance [22] was performed with Phylip (ver. 3.698) using all 26 populations reported in 1000 Genomes plus the Sardinian population. Population relationships were checked via Multidimensional Scaling (MDS), based on the matrix of genetic distances, obtained through Statistica software (ver. 7). The frequency map of the SNP rs143384 (G allele) in the 27 populations was drawn using the software Surfer v. 8.0.

To reveal possible traces of selective pressure, Tajima test and Population Branch Statistic (PBS) were employed. For Tajima test, we used the data of 2504 individuals from 1000 Genomes, while for PBS, we analyzed 307 individuals from the following populations: Northern and Western Europeans in Utah (CEU), Han Chinese in Beijing (CHB), and Yoruba in Ibadan, Nigeria (YRI). An analysis was carried out with specific R packages [23]. Using VCFtools (v 0.1.16) [24], a filter was applied to eliminate all variants with a MAF (Minimum Allele Frequencies) of ≤0.05. PBS was obtained from FST calculated for paired populations (CEU, CHB, and YRI) [25] and plotted using the ggplot2 package in R Studio.

## 3. Results

The genotype and allelic frequencies of SNP rs143384 in the Sardinian sample were calculated with the Genepop Program (ver. 4.4.3). The genotype distribution showed that the most abundant class was represented by the heterozygotes A/G with a frequency of 57.45, while the homozygotes A/A reported a frequency of 28.72, and the homozygotes G/G showed a frequency of 13.83. As for the allelic frequencies, the ancestral allele G showed a frequency of 0.426, and the derived A allele showed a frequency of 0.574. 

When tested for the Hardy–Weinberg equilibrium, Genepop gave a *p* value of 0.969, indicating that the Sardinian population meets the Hardy–Weinberg equilibrium. 

We then compared our data with the worldwide population data reported in the 1000 Genomes database; there is an evident heterogeneous distribution in the allele frequency as well as in the genotype frequency. In particular, allele A in Africa is present at the very low frequency of 3%, whereas in East Asia, the same allele reaches a frequency of 71% (Table 1). Our sampling is within the range of the European variability, where the MAF (G) varies between 35% (CEU, Utah residents with European ancestry) and 47% (Tuscan population). Compared to the only other Italian population (from Tuscany) present in the dataset, the Sardinian population shows just a modest decrease in the frequency of the G allele (42.6% vs. 47.2%). 

**Figure 1:**
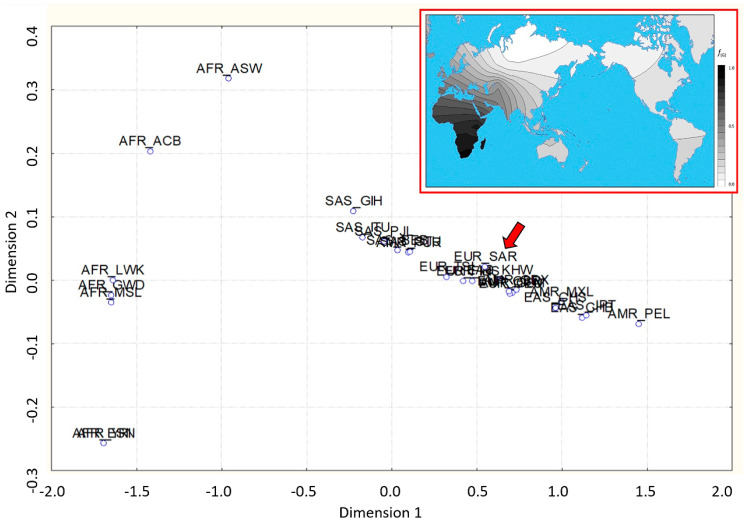
MDS of the rs14334 of the *GDF5*. Population labels according to 1000 Genomes; the red arrow indicates the Sardinian population (labeled EUR_SAR). The box in the upper right corner shows the frequency distribution of the G allele.

The MDS analysis (Figure 1) confirmed the strong differentiation of the African populations from the others; indeed, all of the African populations clustered in a unique area in the first and third quadrants, characterized by negative values of the first component, while the remaining populations occupied a restricted area prevalently in the second and the fourth quadrants. The Sardinian population is placed within the European cluster. Moreover, the African populations occupy a large area of the graphic, suggesting an evident heterogeneity, as expected from the Out of Africa hypothesis. 

Given the remarkable variability in the worldwide frequencies’ distribution of the SNP rs143384, we decided to check for the possible presence of a selective pressure on the gene. For this aim, we used the Tajima and PBS tests, which proved to be the best methods for detecting traces of natural selection in the genome [25]. 

**Figure 2:**
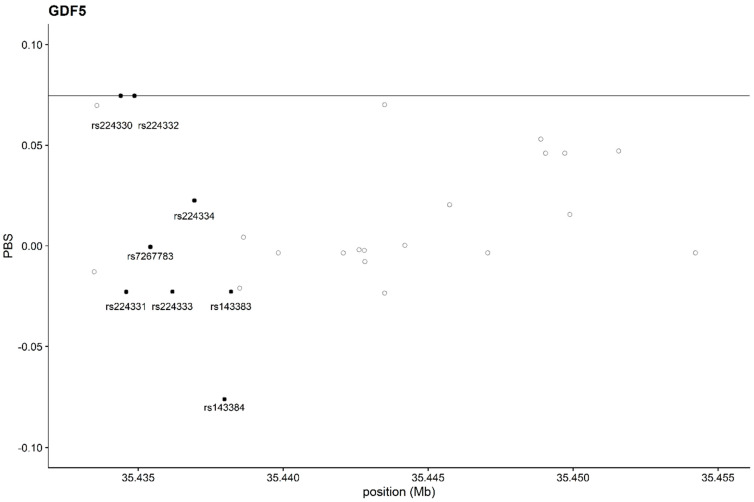
PBS representation of *GDF5* gene. The 99.9th percentiles (continuous horizontal line) of the empirical distribution are shown. Circles represent the SNPs reported in 1000 Genomes; filled circles represent the SNPs in GWASs.

In the plot of the PBS (Figure 2), no outliers among the SNPs in the *GDF5* gene were identified; the two SNPS, rs224330 and rs224332, are located at the top of the empirical distribution of the PBS values for *GDF5*, just on the line of the 99.9th percentile, whilst the two SNPs reported as potentially correlated with osteoarticular diseased or muscular flexibility (rs143384 and 143383) have low PBS values, suggesting that there is no signal of selective pressure in those areas of the genes. 

The Tajima test confirmed what emerged from the PBS analysis; indeed, the Tajima values for each SNP were always positive or around zero, particularly the values for rs143383 and rs143384, which were 2.463 and 0.757, respectively, suggesting that there were no traces of selective sweep.

## 4. Discussion

In the present research, we investigated the possible evolutionary forces that have shaped the frequencies of the polymorphism rs143384 G/A, which is considered a risk factor for osteoarthritis in adults according to several GWASs. 

The worldwide distribution of the rs143384 SNP mirrors the spread of modern humans out of Africa according to the Single Origin Model [26] that originated in Africa about 200 K years ago and then the initial migration to South Asia from 100 K to 70 K years ago, reaching, more recently, Europe and Northeast Asia about 50–45 K years ago, and eventually colonizing the Americas 20–15 K years ago. Indeed, the African population appears almost monomorphic for the ancestral G allele, while the derived A allele appears to be rather frequent in the South Asian regions that were reached by the early expansion of Homo sapiens, with a progressive increase in the areas of more recent colonization such as Europe, East Asia, and the Americas (Table 1). This overall clinal distribution from the ancestral modern human homeland (Figure 1), pointing to an isolation by the distance model, suggests the combined effect of gene flow, genetic drift, and possible founder effects due to subsequent waves of migrations, according to the demic diffusion hypothesis [27]. The Sardinian sample falls within the European variability, not showing any sign of genetic peculiarity in the island population, unlike what was previously observed for other markers [28]. It should be noted that rs143383, the other SNP found to be associated with osteoarthritis via GWAS analysis, shows an analogous worldwide allelic distribution (Table 1).

The knowledge of the genetic variability of *GDF5* polymorphisms, particularly rs143383 and rs143384, is of great importance in evolutionary medicine and biomedical engineering, since genetic and epigenetic manipulations have been suggested as new therapies against OA [13].

Several recent studies, based on GWAS analysis, have suggested that some allelic variants may be associated with several pathologies; therefore, we investigated if their distribution could be the result of a natural selection. 

The search for selective forces within the human genome is becoming increasingly important in evolutionary studies, particularly in evolutionary medicine. It is well known that SNPs related to advantageous or disadvantageous phenotypes can be selected (either fixed or removed) by evolutionary forces. The loss of disadvantageous alleles (negative selection) generally reduces the degree of variance among populations, whilst the fixation of a new variant (positive selection) leads to an increment in population differentiation [29]. 

The Tajima test and the PBS analysis reported here do not show evidence of selective pressure on the whole *GDF5* gene, and in particular, the rs143384 polymorphism considered here falls in the lower percentile with respect to all of the other variants (Figure 2). 

In conclusion, even though the data in the literature show an association with pathological conditions, the allelic variation for rs143384 is consistent with a worldwide distribution that has been shaped by random events following the modern human migration out of Africa. Therefore, the observed variation might be due to genetic drift (including founder effect and/or bottleneck), gene flow, and other demographic changes that the populations may have experienced. Finally, the data reported in the present study are not in contrast with the data reported in the literature; indeed, it is entirely possible that SNP rs143384 may be truly associated with osteoarticular diseases. However, there is no evidence of natural selection, probably because such pathologies are not early onset. Therefore, such variant is expected to have a negligible, if any, effect on the individual fitness, with no apparent consequence on reproductive ability. In this scenario, the distribution of the rs143384 polymorphism is the result of random evolutionary forces.

## Figures and Tables

**Table 1:** Polymorphisms of the *GDF5* gene involved in GWASs on osteoarthrosis. SNP: SNP name; POS: position on build 38 (GRCh38); REG: gene region; REF: reference allele on GRCh38; ALT: most common alternative allele. The frequencies of the reference allele are from the 1000 Genomes database.

SNP	POS	REG	REF	ALT	Africa	Europe	S. Asia	E. Asia	America
rs143383	35438203	5′UTR	G	A	0.9720	0.3649	0.5782	0.2906	0.311
rs143384	35437976	5′UTR	G	A	0.9754	0.4068	0.5865	0.2923	0.345

## Data Availability

The data presented in this study are available upon request from the corresponding authors.
